# The Role of Oxidative Stress and Inflammation in Cardiovascular Aging

**DOI:** 10.1155/2014/615312

**Published:** 2014-07-20

**Authors:** Junzhen Wu, Shijin Xia, Bill Kalionis, Wenbin Wan, Tao Sun

**Affiliations:** ^1^Shanghai Institute of Geriatrics, Department of Geriatrics, Huadong Hospital, Fudan University, Shanghai 200040, China; ^2^Department of Perinatal Medicine, Pregnancy Research Centre and Department of Obstetrics and Gynaecology, University of Melbourne, Royal Women's Hospital, Parkville, VIC 3052, Australia; ^3^Department of Neurology, Zhongshan Hospital, Fudan University, Shanghai 200032, China

## Abstract

Age is an independent risk factor of cardiovascular disease, even in the absence of other traditional factors. Emerging evidence in experimental animal and human models has emphasized a central role for two main mechanisms of age-related cardiovascular disease: oxidative stress and inflammation. Excess reactive oxygen species (ROS) and superoxide generated by oxidative stress and low-grade inflammation accompanying aging recapitulate age-related cardiovascular dysfunction, that is, left ventricular hypertrophy, fibrosis, and diastolic dysfunction in the heart as well as endothelial dysfunction, reduced vascular elasticity, and increased vascular stiffness. We describe the signaling involved in these two main mechanisms that include the factors NF-*κ*B, JunD, p66^Shc^, and Nrf2. Potential therapeutic strategies to improve the cardiovascular function with aging are discussed, with a focus on calorie restriction, SIRT1, and resveratrol.

## 1. Introduction

The average age of the world's population is steadily increasing. For example, in the United States, in 2008, the number of people 65 years or older was 38.9 million, which accounts for 12.8% of the population. By 2030, that number is predicted to rise to 72.1 million, comprising almost 19% of the population [1].

Emerging evidence reveals that the rising epidemic of cardiovascular disease (CVD) is fuelled by obesity, hypertension, and diabetes [2]. However, the aging population is also an independent and cumulatively factor in CVD. Aging results in well-defined phenotypic changes, which render the cardiovascular system prone to disease, even in the absence of traditional risk factors [3]. Intrinsic cardiac aging is defined as the slowly progressive age-dependent degeneration and decline in function that make the heart more vulnerable to stress and contribute to increased cardiovascular mortality and morbidity in the elderly [4]. Remarkably, a host of molecular, cellular, structural, and functional alterations that are operative in accelerated arterial aging have also been implicated in the pathogenesis and progression of arterial diseases.

Considerable published evidence shows that with advanced age, production of ROS significantly increases in both the heart [5] and the vasculature [6]. Indeed, there exists an imbalance between the oxidative and antioxidative system that accompanies aging, which culminates in cardiovascular injury. On the other hand, provoked by a continuous antigenic and oxidative stress, a phenomenon appears in the elderly denoted by “inflammaging”, which is used to elucidate the low-grade chronic inflammatory state in the aged population [7]. A large body of evidence suggests that low-grade chronic inflammation makes aged individuals more susceptible to age-related disease [8–10], especially CVD [11–13]. In this review, insights gained from oxidative stress and inflammation studies may reveal important aspects of the mechanisms of cardiovascular aging and provide rational approaches to develop novel therapies for age-related cardiovascular disease.

## 2. Changes in the Vasculature with Aging

Findings from studies of changes in the vasculature that accompany aging in human and animal models support the concept that aging is an independent risk factor for cardiovascular disease [14–17]. Salient features of age-associated changes in the vascular system include luminal dilation, intimal and medial thickening, vascular stiffening, and endothelial dysfunction [18]. Accumulating evidence shows that even in apparently well-aged populations there is increased large artery thickening and stiffness [19], endothelial dysfunction, and ensuing increases in systolic and pulse pressure. These events precede clinical disease and increase the risk of developing clinical atherosclerosis, hypertension, and stroke [20].

### 2.1. Structural and Functional Changes in the Vasculature with Aging

Age-associated changes in the arterial properties of individuals who are considered otherwise healthy may have relevance to the steep age-dependent increase in vascular diseases. Cross-sectional studies in humans have found that wall thickening and dilatation are prominent structural changes that occur within large elastic arteries during aging [19]. Progressive intima thickening (IM), which can be measured by pulse wave velocimetry (PWV), is usually a risk factor for cardiovascular disease with aging in humans [18]. Furthermore, the degree of IM thickness as a risk factor in other individuals equals or exceeds that of most other conventional risk factors [18, 21]. Other than stiffness, another prevalent vascular alteration is calcification in the aging population [22].

### 2.2. Molecular and Cellular Changes in Vasculature with Aging

Insights gained from cellular and molecular studies in aged vasculature help our understanding of the formation and development of cardiovascular disease. Age-associated remodeling of the walls of large arteries of rodents and nonhuman primates is quite similar to that observed in humans. These changes include luminal dilatation, intimal and medial thickening, vascular stiffening, and endothelial dysfunction [20]. The development of physiological aging is subject to the regulation by signaling pathways, which are influenced by endogenous and exogenous factors. At the cellular level, decreased protein synthesis, increased angiotensin II levels, mitochondrial dysfunction, oxidative stress, and altered patterns of calcium regulation as well as increased DNA, protein, and lipid oxidation are held mainly responsible [23].

The thickened intima in older rats is composed of matrix molecules including collagen, proteoglycans, and vascular smooth muscle cells (SMCs), which migrate into the intima from the media in aged vascular systems. Intimal thickening is the key step in the formation of atherosclerosis. Furthermore, the thickened intima exhibits increased immunostaining for transforming growth factor-*β* (TGF-*β*) and interstitial cell adhesion molecule-1 (ICAM-1) as well as zinc-dependent endopeptidase type-2 metalloproteinase (MMP-2) and its activator, membrane type metalloproteinase-1. TGF-*β*, an important factor for vascular remodeling, is a potent factor in the synthesis of extracellular matrix proteins and is associated with an age-induced increase in arterial fibronectin and collagen [24]. Aging also impairs the intracellular signal transduction system and reduces the activity of nitric oxide (NO), which is derived from the endothelium and exhibits vasodilatory effects [25]. Concurrently, the expression of endothelium-derived NO synthase (eNOS), which influences the production of NO is reduced, whilst expression of cytokines and ICAM increases [26]. Moreover, in aged endothelial cells, increased expression of a cell cycle-controlling factor, senescence-associated *β*-galactosidase (SA*β*gal), as well as telomere shortening and inhibition of telomere activity, have all been observed [27].

## 3. Changes in the Heart with Aging

The heart is a vital organ in the body responsible for pumping blood throughout the circulatory system via continuous rhythmic contractions. The proper functioning of the heart is entirely dependent on a constant supply of oxygen and energy. Therefore, it is not surprising that the heart has a limited life span. Indeed, intrinsic cardiac aging, in the absence of other cardiovascular risk factors, has been shown in many species [3, 28, 29].

There is a continuum of expression of cardiac structural and functional alterations that occurs with age in healthy humans, and these age-associated cardiac changes seem to have relevance to the steep increases in left ventricular hypertrophy (LVH), chronic heart failure, and atrial fibrillation (AF) that are seen with increasing age [30].

### 3.1. Structural and Functional Changes in the Heart with Aging

Increased left ventricle (LV) wall thickness, alterations in the diastolic filling pattern, impaired LV ejection, and heart rate (HR) reserve capacity as well as altered heart rhythm are the most dramatic changes in cardiac function that occur with aging in healthy persons, when assessing cardiovascular function in healthy adult subjects ranging in the age from 20 to 85 years [30].

### 3.2. Cellular and Molecular Changes in Heart with Aging

The heart is comprised of multiple cell types and tissues, in addition to myocytes, that is, interstitial fibroblasts, matrix and arteries. The number of cardiac myocytes becomes reduced as a result of necrosis and apoptosis, along with enlargement of myocyte size. The expression of atrial natriuretic [31] and opioid peptides [32], molecules that are usually produced in response to chronic stress, is increased in the senescent rodent heart. Coordinated changes in the function or expression of proteins that regulate several key steps in the cardiac cell excitation-contraction coupling process occur in the rodent heart with aging and result in a prolonged action potential (AP), a prolonged cytosolic calcium transient after excitation and a prolonged contraction. This altered pattern of Ca regulation and myosin protein expression allows the myocardium of older hearts to generate force and active stiffness for an extensive period after excitation [24].

## 4. Oxidative Stress and Cardiovascular Aging

### 4.1. The Relationship between Oxidative Stress and Cardiovascular Aging

First proposed by Harman, in 1956, the free radical theory of aging postulates that the production of intracellular reactive oxygen species is the major determinant of lifespan [33]. Currently, oxidative stress and impaired antioxidant defense mechanisms are believed to be key contributors to the cardiovascular aging process [34]. Oxidative stress develops as a consequence of excessive generation of reactive oxygen species (ROS), by enzymes such as NADPH oxidase, uncoupled nitric oxide synthase, and xanthine oxidase, by the mitochondrial electron transport chain, and as a result of reduced antioxidant capacity [35]. In the vessel wall, the majority of cellular ROS are generated by the mitochondrial oxidative phosphorylation system, which is a vital component of the mitochondrial free radical theory of aging [36]. In the senescent heart, the majority of ROS are derived from NADPH oxidase and the mitochondrial electron transport chain (ETC) [29]. There is substantial evidence supporting the involvement of oxidative stress in the genesis of vascular damage within the aging process. Increased oxidative stress and ROS production, associated with decreased endothelial NO bioavailability, have been detected during the aging process in different vascular beds in various animal models including the rat aorta [37], coronary arteries [38], and mouse aorta [39]. Elevated oxidative stress in the senescent myocardium has several consequences such as enhanced protein oxidation/nitration, reduced bioavailability, lipofuscin formation, activation of inflammatory response, antioxidative stress response, apoptosis, and endoplasmic reticulum (ER) stress [29]. Importantly, superoxide anion (O_2_^−^) reacts with endothelium-derived NO to form peroxynitrite (ONOO^−^), a potent nitrating and oxidizing agent. There is a growing body of evidence that demonstrates substantially enhanced cardiovascular peroxynitrite (OONO^−^), superoxide anion (O_2_^−^) formation, and decreased NO bioavailability, with aging. Experimental studies show that aging leads to reduced NO production and endothelial dysfunction, regardless of other risk factors [25, 39]. Moreover, another theory posits that the endothelium of aged subjects produces enough NO to reach full relaxation of the mesenteric vasculature, but this NO is partially counteracted by COX-derived vasoconstricting compounds and reactive oxygen species, probably superoxide anions, which are also produced by endothelial cells [40]. In any case, increased ROS production ultimately initiates the endothelial dysfunction and myocardium damage. Impaired endothelial vasodilation is early manifestations of arterial aging. These impaired processes precede, by years, the clinical manifestations of vascular dysfunction, which are the first step toward cardiovascular disease and influence vascular outcome in the elderly [41].

NO, produced by endothelial NO synthase (eNOS), is the most important vasodilator and is a marker of vascular health. Impaired NO bioavailability is observed in hypertension, diabetes, and atherosclerosis [42]. Aging may impair eNOS functionally via several mechanisms. During the aging process in rat arteries, the expression of eNOS is lowered, resistance to oxidation is weakened, and the response of vascular smooth muscle to NO is apparently decreased [43]. The decreased expression of eNOS that accompanies aging is attributed partly to L-arginine, a major eNOS substrate, which is degraded by arginase II [44]. Interestingly, arginase II activity and expression increase with age, resulting in decreased eNOS availability and reduced NO synthesis and thus contributes to endothelial dysfunction. Besides L-arginine, tetrahydrobiopterin (BH4) is an important cofactor for eNOS activity, which is involved in age-related endothelial dysfunction [44]. Indeed, increased oxidative stress rapidly degrades BH4 leading to altered eNOS efficiency whereas exogenous administration improves endothelium-dependent vasodilation in human aging [45]. Worthy of note is that age-related eNOS dysfunction is critically involved in microvascular dysfunction and impaired ventricular contractility in elderly patients [46].

## 5. Antioxidant Response in Cardiovascular Aging

As previously stated, oxidative stress may result from increased ROS generation, a defective antioxidant defense system, or both.

### 5.1. Nrf2 Signaling Associated with Cardiovascular Aging

Among transcription factors involved in the cellular response to ROS generation, nuclear factor erythroid-2 related factor-2 (Nrf2) is an evolutionarily highly conserved redox-sensitive transcription factor that is activated by ROS production in the vasculature of young animals, leading to the upregulation of various antioxidant genes [47]. Despite an increase in superoxide production, aortas from Fischer 344 × Brown Norway rats show that aging results in a progressive increase in O_2_^−^ production, as well as downregulation of protein and mRNA expression of Nrf2. Consequently, there is decreased nuclear Nrf2 activity and decreased expression of Nrf2 target genes (i.e., NADPH quinine oxidoreductase-1, glutamylcysteine synthetase, and heme oxygenase-1) [48]. In aging vessels, increased production of ROS fails to activate Nrf2, resulting in increased blood vessel sensitivity to the deleterious effects of ROS [48]. An impairment of the Nrf2 system could contribute to the reduced angiogenic capacity of aged vasculature and its defective response to ischemic injuries, since disruption of Nrf2 signaling impairs angiogenic processes in human coronary arterial endothelial cells [49]. Vascular Nrf2 dysfunction associated with aging would exacerbate cellular oxidative stress and increase the sensitivity of aged vessels to cellular damage. Interestingly, Nrf2 dysfunction exerts proinflammatory effects by exacerbating ROS-mediated NF-*κ*B activation (as described below) in aging [47]. Thus, aging is associated with Nrf2 dysfunction in the vasculature, which likely exacerbates age-related cellular oxidative stress and increases sensitivity of aged vessels to oxidative stress-induced cellular damage.

### 5.2. Superoxide Dismutase Activity

Despite a higher capacity of ROS production in the heart, this organ is well equipped with antioxidant enzymes capable of scavenging free radicals. However, the literature has been controversial about the changes that occur in the antioxidant reserve during cardiac aging, with some studies showing enhanced content and activity [5], whilst others reported no change [50]. An efficient defense mechanism against superoxide radicals produced during oxidative stress is provided by the activity of superoxide dismutase (SOD). The three distinct isoforms of SOD, the cytosolic copper-zinc SOD (Cu/Zn SOD, SOD-1), mitochondrial manganese (MnSOD, SOD-2), and extracellular SOD (EcSOD, SOD-3), have evolved as the key enzymatic system for converting oxygen radicals to hydrogen peroxide and molecular oxygen [51]. MnSOD is the main antioxidant enzyme that scavenges superoxide anions in the inner mitochondrial matrix and acts as a first line of defense against mitochondrial oxidative stress [52]. Studies conducted in MnSOD-deficient mice demonstrate the detrimental effect of superoxide anions and therefore of ROS in mitochondria [53]. Several studies reported decreased expression of MnSOD antioxidant enzyme in aortas of old mice [54]. In humans, while some authors have not observed an alternation in MnSOD expression in endothelial progenitor cells with aging [55], others have demonstrated a decrease of this enzyme's expression in endothelial cells of sedentary aged subjects [56]. The role of Cu/ZnSOD is to limit the increase in superoxide and therefore to maintain normal endothelial vasodilation in vascular vessels. In humans, a reduction in Cu/ZnSOD protein expression and total SOD enzymatic activity were observed in aged mesenteric lymphatic vessels [57]. The ecSOD is the major SOD isoform in the vascular extracellular space, thereby protecting it against NO inactivation by free radicals during its diffusion to smooth muscle [58]. Reduced mRNA levels of ecSOD, but not Cu/ZnSOD or MnSOD, were reported in aged mice when compared with younger mice [59].

## 6. Gene Regulation of Transcription

### 6.1. JunD Signaling Is Involved in Endothelial Dysfunction

Activator protein-1 (AP-1) is a collection of dimeric complexes made by different members of three families of DNA-binding proteins; Jun, Fos, and ATF/CREB [60]. JunD is the most recently discovered gene of the Jun family. JunD regulates cell growth and survival and protects against oxidative stress by modulating genes involved in antioxidant defense and ROS production [61]. There is a conspicuous link between the deletion of the JunD gene, ROS generation, and endothelial dysfunction. Evidence has been presented for increased ROS generation in immortalized JunD^−/−^ cells [62]. Furthermore, gene expression profiling of JunD^−/−^ cells showed downregulation of several free radical scavenging enzymes associated with an increase in the expression of ROS-producing NADPH oxidase [63]. Interestingly, evidence that JunD may be a critical regulator of vascular homeostasis was obtained by investigating its role in ROS-driven vascular aging [63]. Young JunD^−/−^ mice showed an impairment of endothelium-dependent relaxation, as a result of acetylcholine stimulation, that was similar to that observed in aged WT mice, suggesting premature endothelial aging in animals lacking the AP-1 transcription factor JunD. Moreover, JunD overexpression performed by intravenous injection of a predesigned JunD cDNA clone improved acetylcholine-dependent relaxation compared with vector-treated mice. Therefore, aged mice showed a downregulation of JunD expression compared with younger animals. Together with diminished expression, it was also reported that JunD transcriptional activity is reduced in aged vessels [63]. Oxidative stress in JunD^−/−^ mice was associated with early features of vascular aging, including reduced telomerase activity, increased *β*-galactosidase staining, and upregulation of the senescence markers p53 and p16^INK4a^ [63]. Finally, JunD protein levels are decreased in patients with end-stage heart failure suggesting that the transcription factor may protect against age-related cardiac dysfunction [64].

### 6.2. The p66^Shc^ Signaling Pathway in Endothelial Dysfunction

p66^Shc^ functions in the intracellular pathway that converts intracellular oxidative signals into apoptosis. Multiple lines of evidence implicate p66^Shc^ in aging and in the pathogenesis of aging-associated diseases in mammals [39]. Intracellular free radicals are reduced in cells lacking the p66^Shc^ gene (p66^Shc−/−^ cells), and both systemic as well as intracellular free radicals are diminished in p66^Shc−/−^ mouse models exposed to high oxidative stress [65]. Accordingly, mice lacking the p66^Shc−/−^ gene display a prolonged lifespan as well as increased resistance to oxidative stress and apoptosis. p66^Shc−/−^ signaling is required to induce a ROS-driven vascular senescent phenotype [39]. Importantly, p66^Shc^ activation is thought to be upstream of NADPH and to be a mammalian target of the rapamycin (mTOR) pathway, two important determinants of vascular damage [66]. p66^Shc−/−^ mice showed increased NO bioavailability and decreased production of superoxide O_2_^−^, compared with WT aged mice [39]. Indeed, diabetic p66^Shc−/−^ mice were protected against myocardial oxidative stress, apoptosis, and telomere shortening [67]. Moreover, ablation of the p66^Shc^ gene in cardiac stem cells preserved the growth reserve of the heart [67]. The clinical relevance of p66^Shc^ is supported by the notion that p66^Shc^ gene expression is increased in mononuclear cells obtained from patients with type 2 diabetes and coronary artery disease [68]. Most important is that p66^Shc^ is part of a signal transduction pathway relevant to endothelial integrity. The long-lived p66^Shc−/−^ mice are protected against age-related endothelial dysfunction [39].

## 7. Inflammation in Cardiovascular Aging

ROS, as mentioned above, is produced increasingly with age as a result of a variety of stimuli including physical, chemical, and biological agents. These factors predispose to endothelial dysfunction and cellular damage that lead to the process of aging. Interestingly, emerging evidence provides a close link between oxidation and inflammation, since excessive or uncontrolled free radical production can induce an inflammatory response and free radicals are inflammation effectors [69]. Indeed, a low level of chronic inflammation is associated with most age-related diseases including atherosclerosis, cardiovascular diseases, and diabetes [70]. This phenomenon is provoked by continuous antigenic load and stress and is accompanied by a global reduction in the capability to cope with a variety of stressors as well as a concomitant, progressive increase in proinflammatory status [7]. The term “inflammaging” was coined by Franceschi et al. to denote the upregulation of the inflammatory response with progressing old age and the ensuing low-grade chronic systemic proinflammatory state that underlies most age-associated diseases [7].

### 7.1. The Role of Inflammation in Cardiovascular Aging

Aging is accompanied by immune, hormonal, and adipose changes leading to a chronic inflammatory state. Inflammation may constitute a biological foundation for the pathophysiological process of frailty. These changes influence the onset of frailty and cognitive decline as well as cardiological, neurological, and vascular events. However, inflammation is not a negative phenomenon* per se, *since it is needed to maintain life through a constant struggle to preserve the integrity of the individual. Importantly, if the levels of inflammatory compounds exceed the control of anti-inflammatory compounds, an imbalance occurs and an inflammatory state is established [70]. Still under debate is the precise cause that underlies the low-grade inflammatory process associated with aging which leads to the development of the age-related inflammatory chronic diseases such as atherosclerosis [71] and hypertension [72]. A current hypothesis is that chronic stimulation of the immune system contributes to the proinflammatory shift [73, 74]. Multiple lines of evidence demonstrate that aging is commonly accompanied by a progressive deregulation of the immune response, mainly due to alterations of the cellular/adaptive immune response, especially T cell responses [73]. Compared with these changes in cellular immunity, some features of innate immunity are relatively well maintained with age [73]. Thus, this apparent disequilibrium between the retention of relatively reactive innate immune response with aging leads to the presence of a low-grade inflammatory status commonly present in the elderly. The cause of this is certainly multifactorial. One of the likely principal causes is chronic antigenic stimulation by cytomegalovirus (CMV), bacteria, and other viruses or endogenous cellular factors such as posttranslationally modified macromolecules, including DNA, or proteins that can be modified or continuously released from tissues such as the elastin peptides (EPs) [71]. These modifications may also result in chronic stimulation of adaptive immune responses, recognized by an inverted CD4 : CD8 ratio that is caused by an overwhelming expansion of CD8 + cells [74]. The processes described above are thought to culminate in the chronic low-grade inflammatory process that accompanies aging.

Low-grade systemic inflammation characterized by the elevation of circulating acute-phase proteins and proinflammatory cytokines is associated with frailty as well as the development and progression of severe, age-related conditions such as cardiovascular disease (CVD). Atherosclerosis/atherothrombosis is the major cause of the rising epidemic of CVD. Just three decades ago, atherosclerosis was envisaged as a bland proliferative process [75]. Multiple independent pathways of evidence now pinpoint inflammation as a key regulatory process that links multiple risk factors for atherosclerosis and its complications with altered arterial biology [76]. The endothelium, the inner lining of all blood vessels, maintains homeostasis through a balance of endothelium-derived factors. Disruption by inflammatory and traditional cardiovascular risk factors leaves the vasculature susceptible to atherogenesis, the process of forming plaques in arteries. Inflammatory mediators play a fundamental role in the initiation, progression, and eventual rupture of atherosclerotic plaques. TNF-*α* activates a proinflammatory gene expression profile in endothelial cells that promotes adherence of monocytes and their migration into the subendothelial layer. At a molecular level, E-selectins mediate leukocyte rolling, whereas chemokines lead to leukocyte activation, whilst intercellular adhesion molecule-1 and vascular cell adhesion molecule-1 contribute to leukocyte adhesion. In the vasculature, monocytes undergo transformation into macrophages that internalize modified lipoproteins and give rise to foam cells. In parallel, upregulation of hemostatic proteins induces a highly procoagulative state of activated endothelium. The evolution of a fatty streak towards a complex atherosclerotic lesion is characterized by amassed oxidized low-density lipoproteins (LDLs) exerting toxic effects on macrophages and smooth muscle cells (SMCs), which culminates in the formation of a necrotic core [77]. In this process, SMCs migrate from the* tunica media* into the* intima* via degradation of the extracellular matrix, which is mediated by MMP-9 as well as other proteinases [78]. In the* intima*, SMCs proliferate under the influence of various growth factors and secrete extracellular matrix proteins, including interstitial collagen, especially in response to transforming growth factor-*β* (TGF-*β*) and platelet-derived growth factor. These molecular processes cause the lesion to evolve from a lipid-rich plaque to a fibrotic and then calcified plaque, which may create a stenosis [79]. Acute thrombotic complications such as atherothrombosis can result in vessel occlusion and the restriction in blood flow may trigger the manifestation of disease, heart attacks. We have emphasized that endothelial dysfunction is an early hallmark of vascular disease that can occur well before the structural manifestation of atherosclerosis. Endothelial dysfunction can serve as an independent predictor of future cardiovascular episodes and we highlighted the vital role that inflammation plays in initiating many of the adverse events. Indeed, increased arterial stiffness, as revealed by a greater pulse pressure and pulse wave velocity (PWV), has been associated with many cardiovascular risk factors and is reflected by raised inflammatory biomarkers such as high C-response protein levels [21]. Notably, increasing evidence links inflammatory processes to atherogenesis. Markers of inflammatory activation and endothelial dysfunction provide useful information about a patient's risk and stage of developing cardiovascular disease, as described below.

In humans, considerable evidence supports that essential hypertension is a condition of chronic low-grade inflammatory status [80, 81]. Evidence of immune system involvement in the vasculature from patients with hypertension or cardiovascular disease is beginning to appear in the literature. Inflammation participates in many processes that contribute to the development of elevated blood pressure (BP). In the vasculature, inflammation can enhance the proliferation of smooth muscle cells and play a role in vascular remodeling [80]. In animal models of hypertension, there is evidence of infiltration of inflammatory cells from the innate immune system (i.e., dendritic cells, NK cells, monocytes, and macrophages) in perivascular fat and adventitia of blood vessels as well as in other target organs (i.e., kidney and heart) [81]. Osteopetrotic mice have a mutation in the macrophage colony-stimulating factor (Csf1) gene and thereby have macrophages that are functionally deficient. Osteopetrotic mice remain normotensive and develop less endothelial dysfunction, vascular remodeling, and oxidative stress, despite infusion of angiotensin II (Ang-II) [82] or deoxycorticosterone acetate (DOCA) salt treatment [83], compared with wild-type littermates. Together, these studies suggest a role for macrophages in the pathogenesis of hypertension and vascular damage. Monocytes and macrophages express functional Ang-II and mineralocorticoid (MR) receptors, the activation of which lead to ROS generation via activation of NADPH oxidase and cytokines [13]. Monocyte and macrophage-derived vascular ROS may upregulate the expression of chemokines and adhesion molecules, reduce NO bioavailability, stimulate VSMCs hypertrophy, and activate matrix metalloproteinases leading to vascular dysfunction and remodeling [84, 85]. Furthermore, Treg cells involved in adaptive immunity produce IL-10, a significant anti-inflammatory cytokine that exerts an effect on the improvement of microvascular endothelial function in hypertension [72]. Aortic stiffness, measured by carotid-femoral PWV, constitutes a hallmark of the aging process and is an independent predictor of adverse cardiovascular events in hypertension [86]. Indeed, low-grade inflammation in conjunction with hypoadiponectinemia exerts an additive detrimental effect on aortic stiffness thereby accelerating the vascular aging process [87]. However, it is not evident whether vascular inflammation causes the arterial stiffening and hypertension or it is a consequence of high blood pressure stiffening the arteries that favors a cascade which culminates in vascular inflammation and increased arterial stiffness.

Arterial fibrillation (AF) is the most common cardiac arrhythmia and its prevalence in the general population is increasing rapidly [88]. An analysis from the Framingham Heart Study reports that the associations could be due to in part the well-established associations between inflammation and prevalent cardiovascular conditions that predispose to AF [89]. Conen et al. provide evidence that inflammation, which is measured by plasma levels of high-sensitivity C-reactive protein, sICAM-1, and fibrinogen, is significantly associated with AF events in a female population without a history of cardiovascular disease and where traditional risk factors were controlled for [90]. These findings suggest that inflammation may be involved in the pathogenesis of AF. A substantial body of evidence reveals that patients with rheumatoid arthritis (RA) have an increased risk of coronary heart disease, an increased standardized mortality ratio, and a shortened life expectancy by 3–18 years when compared with the matched non-RA population [91]. An abundance of data identifies enduring systemic inflammation as the pathophysiological basis for linking RA to accelerated heart disease development. Although the specific pathophysiological link between systemic inflammation and cardiovascular disease is not completely clear, the promotion of accelerated coronary atherosclerosis is considered the most important mechanism for the higher prevalence of ischemic heart disease (IHD) in RA [92]. In fact, systemic release of proinflammatory cytokines (IL-1, IL-6, and TNF-*α*) in RA synovial tissue could boost the immunoinflammatory process underlying atherogenesis either directly by affecting the cells of the plaque or indirectly by stimulating a number of proatherogenic functions of liver, adipose tissue, skeletal muscle, and vascular endothelium [93]. A plethora of data investigating HIV-positive patients demonstrates increased coronary arterial disease rates compared with a non-HIV population, particularly among women [94, 95]. Nordell et al. [96] assessed the prognostic value of inflammatory and coagulation markers with respect to fatal outcomes among patients with HIV who experience CVD events. Their findings suggest that chronic inflammation and activated coagulation associated with HIV lead to a poor outcome when CVD event occurs.

### 7.2. Predictors of Cardiovascular Disease That Are Involved in Inflammation

As mentioned above, inflammation plays an important role in cardiovascular disease. The immune system produces more proinflammatory cytokines under the chronic stimulus that accompanies aging. Knowledge has flourished regarding the predictive value of several inflammatory markers, on the incidence of cardiovascular events, even in the apparently well healthy population [97–99]. Among the inflammatory markers, interleukin-6 (IL-6), C-reactive protein (CRP), and TNF-*α* have generated considerable attention. Over 15 years ago, an association between enhanced inflammations, as demonstrated by higher plasma levels of CRP in middle-aged men without previous cardiovascular disease, was reported using data from the Multiple Risk Factor Intervention Trial (MRFIT) [100]. Low-density lipoprotein (LDL) is the focus of current guidelines for the determination of the risk of cardiovascular disease [101]. Ridker et al. [102] conducted a study to determine whether CRP is a stronger predictor of future cardiovascular events than LDL cholesterol. Findings from the Health ABS Study [103] indicate that IL-6, TNF-*α*, and CRP markers predicted the onset of cardiovascular events. Individuals with high levels of the three markers had the greatest risk of cardiovascular events. Moreover, increased IL-6 levels were the strongest and most consistent risk factor for cardiovascular events. According to this study, IL-6 and TNF-*α* showed more consistent results than CRP in predicting cardiovascular events. Concomitantly, it was suggested that TNF-*α* and IL-6 are associated with the severity of left ventricular dysfunction and with the degree of activation of the sympathetic and renin-angiotensin systems [11]. In humans, many lines of evidence that established essential hypertension as a condition of chronic low-grade inflammatory status also revealed a strict and independent association between CRP, TNF-*α*, and IL-6 or adhesion of molecules and vascular changes in essential hypertensive patients [72]. Therefore, HIV-positive patients with higher levels of inflammatory markers, that is, IL-6 and hsCRP, showed an increased risk of more fatal CVD events [96]. Worth noting is that further studies are needed to determine whether inflammatory markers might represent valid targets for new medications that modify the atherosclerosis process and prevent cardiovascular disease.

### 7.3. The Inflammatory Phenotype of Senescent Cells

As stated above, a common feature of aging tissues is low-grade chronic inflammation, termed “inflammaging” that may derive partly from an age-related decline in homeostatic immune function or resistance to endogenous microbes. Nevertheless, chronic inflammation may also derive in part from senescent cells, and proteases, termed the senescence-associated secretory phenotype (SASP). The SASP, through the inflammatory, growth-promoting, and remodeling factors that it produces, can potentially explain how senescent cells alter tissue microenvironments, attract immune cells, and unexpectedly induce malignant phenotypes in nearby cells [103]. Proteins that are associated with the SASP, such as TNF-*α*, IL-6, MMPs, monocyte chemoattractant protein-1(MCP-1), and IGF binding proteins (IGFBPs), increase in multiple tissues with chronological aging and occur in conjunction with systemic inflammation [104].

Endothelial dysfunction, vascular smooth cell (VSMC) proliferation/invasion/secretion, matrix fragmentation, collagenization, and glycation are characteristics of an age-associated arterial phenotype that creates a microenvironment enriched in reactive oxygen species (ROS) needed for the pathogenesis of arterial disease. This niche creates an age-associated arterial secretory phenotype (AAASP), which is orchestrated by the concerted effects of numerous age-modified angiotensin II signaling molecules. The arterial wall is remodeled by the joint effects of fluctuating levels of numerous proteins [105]. Age dramatically alters the volume and contents of the arterial* intima* in rats, nonhuman primates, and humans [18], which was described above. Small, disoriented vascular smooth muscle cells (VSMCs) and collagen type I and type III markedly increase within the thickened intimae of old rats [106]. Molecular elements of the Ang-II signaling cascade are upregulated in aged arterial walls and play a causal role in arterial aging and in vessel disease [27]. The levels of transcription, translation, and activity of MMP-2 are enhanced within the arterial wall with aging [20]. TGF-*β*, VSMCs, and senescence-associated *β*-gal activity also vary with aging. The novel AAASP concept provides an explanation for the observation that the arterial wall of younger animals, in response to experimental induction of low-grade chronic inflammation by hypertension or early atherosclerosis, is transformed into a phenotype that is strikingly similar to that which develops during aging [107].

### 7.4. NF-*κ*B Signaling Regulation in Cardiovascular Aging Associated with Inflammation

NF-*κ*B, an important transcription factor, is regarded as a molecular switch of inflammatory pathways. It is responsible for regulating the gene expression of factors that control cell adhesion, proliferation, inflammation, redox state, and tissue specific enzymes [108]. The NF-*κ*B signaling pathway may regulate inflammaging [109]. Indeed, activation of NF-*κ*B mediates vascular and myocardial inflammation in metabolic and age-related diseases [110]. However, the longevity gene, SIRT1, can be combined with a subunit of NF-*κ*B, Rel/p65 to generate K310 deacetylase, which inhibits the transcriptional activity of NF-*κ*B [111, 112]. NF-*κ*B can enforce aging, whereas SIRT1 may regulate NF-*κ*B to delay aging [113]. NF-*κ*B can regulate both aging and inflammation [114] and it can also inhabit inflammatory reaction by regulating SIRT1 (Sir2 homolog) and FoxO (DAF-1) [109].

MicroRNAs (miRs) are a broad class of small, noncoding RNAs that have revolutionized our understanding of gene transcription and translation. Recent data show that miR regulation entails far more complex posttranscriptional control, with the ability to both repress and activate gene expression by interacting with complementary sequences in coding and noncoding regions of their mRNA targets [115]. Moreover, most of the miRs targeting the NF-*κ*B pathway and its modulators affect NF-*κ*B signaling dynamics primarily through a negative feedback loop aimed at restraining the excessive proinflammatory response induced by signaling activation [116]. An altered expression of the miRs targeting the NF-*κ*B pathway may thus contribute to the dysregulation of the inflammatory/anti-inflammatory balance, promoting carcinogenesis [117]. Interestingly, it has recently been observed that miRs can act as agonists of single-stranded RNA-binding Toll-like receptors (TLRs) both in NF-*κ*B signaling activation and interleukin secretion, thus triggering a proinflammatory response that can promote the creation of a microenvironment favorable to cancer development [118]. Therefore, it is possible that senescent cells contribute to inflammation not only by producing proinflammatory and proangiogenic molecules typical of SASP but also by transferring miRs into other proinflammatory cells, namely, macrophages [119].

In addition, a recent study clearly showed that endothelial suppression of NF-*κ*B prolongs the lifespan of mice and ameliorates obesity-induced endothelial insulin resistance [120]. Impaired insulin signaling is indeed an important hallmark linking metabolic disease with premature aging of the cardiovascular system [121]. Moreover, age-dependent NF-*κ*B activation was associated with systemic inflammation and impaired endothelial dependent vessel dilation [122]. All these finding validate NF-*κ*B as a therapeutic target to prevent cardiac disease in elderly.

## 8. Therapeutic Implications Involved in Inflammation and Oxidation

### 8.1. Caloric Restriction

Caloric restriction (CR) is a dietary regimen, which improves health and slows the aging process in evolutionarily distant organisms by limiting dietary energy intake [123]. There is increasing epidemiological and experimental evidence that CR plays an important role in vasoprotection in aging and in pathological conditions associated with accelerated vascular aging [124]. The mechanisms underlying the beneficial cardiovascular effects of CR are multifaceted and include normalization of mitochondrial biogenesis, attenuation of mitochondrial ROS production, increased bioavailability of NO, and consequential inhibition of signaling pathways regulated by mitochondria-derived ROS. The cellular pathways involved in mitochondrial protection induced by calorie restriction appear to depend on increased expression/activity of the NAD^+^-dependent histone deacetylase SIRT1 [111]. Expression of SIRT1 in mice confers vasoprotection, reduces endothelial ROS production, inhibits NF-*κ*B signaling, and attenuates vascular inflammation, thus mimicking the effects of CR [125]. Furthermore, evidence supports that aortic mTOR signaling was increased with aging in mice but maintained at young adult levels with CR [126]. In addition, CR can also activate the transcription factor Nrf2, which controls the expression of numerous ROS detoxifying and antioxidant genes involved in regulation of mitochondrial redox homeostasis [127].

A recent study of mice demonstrated that CR can prevent, or significantly lessen, multiple adverse features of arterial aging, as well as age-related increases in arterial blood pressure [126]. The research extended insight into the physiological benefits of CR by providing the first direct evidence that CR partially, or completely, prevents large elastic artery stiffening, wall hypertrophy, endothelial dysfunction, reduction in NO bioavailability and increases arterial blood pressure associated with aging in mice [126].

### 8.2. SIRT1

Increasing evidence shows that sirtuins, a conserved family of proteins, mediate a large number of the beneficial effects of CR [126, 128]. Sirtuins function as NAD^+^-dependent deacetylases, which are also called class III histone deacetylases (HDAC). SIRT1 in mammals, the closest homologue of the yeast Sir2 protein, is considered to be a novel antiaging protein involved in the regulation of cellular senescence/aging and inflammation. The mechanism of SIRT1-mediated protection against inflammaging involves the regulation of inflammation, premature senescence, telomere attrition, senescence-associated secretory phenotype, and DNA damage response [129]. SIRT1 upregulation, induced by CR, was also attenuated in eNOS^−/−^ mice, which indicated that NO derived from eNOS played an important role in SIRT1 expression [130]. Indeed, eNOS acts as a direct substrate for SIRT1. In summary, SIRT1 deacetylates eNOS and increase its activity. The inference is that mechanisms of SIRT1 antiaging effect lie partly in its beneficial role of reducing oxidative stress and improving endothelial function. As explained above, SIRT1 can directly inhibit the activity of NF-*κ*B signaling by deacetylating RelA/p65 [111]. Worth mentioning is that the aggravated inflammation in vascular aging was inversely related to the expression of SIRT1, whereas CR upregulated SIRT1 expression [131]. There is confirmation that SIRT1 modulates vascular cell senescence during vascular aging [132]. Indeed, senescent endothelial cells were observed in the coronary arteries of patients with ischemic heart disease. The inhibition of SIRT1 by sirtinol leads to increased p53 acetylation, increased PAI-1 expression, and decreased eNOS activity in HUVECs and induces senescence-like behavior including flattened and enlarged cell morphology, increased SA-*β*-gal activity, and growth arrest [132]. The exacerbated senescence was accompanied by SIRT1 downregulation, increased p53 acetylation, and p21 expression. In addition, SIRT1 overexpression attenuated high-glucose-induced senescence in HUVECs, whereas SIRT1 inhibition had the opposite effect [132]. Interestingly, SIRT1 can also affect atherosclerosis. Worthy of note is that the endothelium-specific overexpression of SIRT1 decreased atherosclerosis in apoE^−/−^ mice [133]. Taken together, improving the function of the cardiovascular system by modulating SIRT1's activity is vitally important. Clinical trials of SIRT1 and its activators will help to bring this promising therapeutic target to reality.

### 8.3. Resveratrol, Activator of SIRT1

Resveratrol, a diet-derived polyphenol, is a prototype of a new class of drugs referred to as CR mimetics, which are being developed to reverse organ pathologies associated with aging and metabolic diseases [134]. The “French paradox,” which describes the phenomenon that morbidity and mortality of coronary artery disease are low in southern France and other Mediterranean territories, is at least partly attributable to resveratrol, which is an important constituent of Mediterranean diets and is involved in vasculoprotection [135]. Resveratrol is thought to have diverse antiatherogenic activities, such as the inhibition of LDL oxidation and platelet aggregation and regulation of vascular smooth muscle proliferation [136, 137]. Multiple lines of evidence indicate that resveratrol inhibits endothelial activation and monocyte adhesion and attenuates proinflammatory gene expression by inhibition of NF-*κ*B activation in coronary arterial endothelial cells [138]. Indeed resveratrol, a SIRT1 activator, was associated with upregulation of eNOS and induction of specific mitochondrial biogenesis factors, which exert a vasoprotective effect [139]. These studies raise the possibility that resveratrol supplementation may confer significant vasoprotection in elderly humans.

## 9. Conclusion

With a keen awareness that aging is an independent risk factor for cardiovascular disease, the means by which to achieve successful cardiovascular aging whilst decreasing the risk of CVD is a worthy pursuit. Oxidative stress and inflammation play a vital role in the process of cardiovascular aging. Endothelial dysfunction that is associated with oxidative stress and inflammation is a fundamental feature of CVD. Moreover, the relationship between oxidative stress and inflammation is bidirectional. A better understanding of the molecular and cellular mechanisms underlying cardiovascular aging, as well as their potential interactions, will provide a growing list of potential targets for specific interventions aimed at preventing or delaying the cardiovascular dysfunction associated with aging. Breakthroughs in achieving successful cardiovascular aging that focus on oxidative stress and inflammation are on the horizon.
